# Metagenomic insights into the wastewater resistome before and after purification at large‑scale wastewater treatment plants in the Moscow city

**DOI:** 10.1038/s41598-024-56870-0

**Published:** 2024-03-15

**Authors:** Shahjahon Begmatov, Alexey V. Beletsky, Alexander G. Dorofeev, Nikolai V. Pimenov, Andrey V. Mardanov, Nikolai V. Ravin

**Affiliations:** 1 Institute of Bioengineering, Research Center of Biotechnology of the Russian Academy of Sciences, Leninsky Prosp, bld. 33‑2, Moscow, Russia 119071; 2 Winogradsky Institute of Microbiology, Research Center of Biotechnology of the Russian Academy of Sciences, Leninsky Prosp, bld. 33‑2, Moscow, Russia 119071

**Keywords:** Antimicrobials, Microbial communities, Industrial microbiology

## Abstract

Wastewater treatment plants (WWTPs) are considered to be hotspots for the spread of antibiotic resistance genes (ARGs). We performed a metagenomic analysis of the raw wastewater, activated sludge and treated wastewater from two large WWTPs responsible for the treatment of urban wastewater in Moscow, Russia. In untreated wastewater, several hundred ARGs that could confer resistance to most commonly used classes of antibiotics were found. WWTPs employed a nitrification/denitrification or an anaerobic/anoxic/oxic process and enabled efficient removal of organic matter, nitrogen and phosphorus, as well as fecal microbiota. The resistome constituted about 0.05% of the whole metagenome, and after water treatment its share decreased by 3–4 times. The resistomes were dominated by ARGs encoding resistance to beta-lactams, macrolides, aminoglycosides, tetracyclines, quaternary ammonium compounds, and sulfonamides. ARGs for macrolides and tetracyclines were removed more efficiently than beta-lactamases, especially *ampC*, the most abundant ARG in the treated effluent. The removal efficiency of particular ARGs was impacted by the treatment technology. Metagenome-assembled genomes of multidrug-resistant strains were assembled both for the influent and the treated effluent. Ccomparison of resistomes from WWTPs in Moscow and around the world suggested that the abundance and content of ARGs depend on social, economic, medical, and environmental factors.

## Introduction

The spread of antimicrobial resistance (AMR) in the environmental microbiome has become one of the frequently noted problems in recent years, along with global climate change, food security and other technological challenges. Numerous studies show that from year to year, in addition to increasing the cost of hospitalization and treatment of patients infected with multidrug-resistant bacteria, the number of deaths of such patients is growing^1,2^. Understanding the mechanisms underlying the emergence, selection and dissemination of AMR, and antibiotic resistance genes (ARGs), is required for the development of sustainable strategies to control and minimize this threat. The dissemination of antibiotic resistant bacteria (ARB) and ARGs occurs differently and this process is more active in urban territories rather than in rural ones. The rate of spread of ARGs and ARB in urban areas is obviously determined by the high population density and, as a rule, wastewater which flows from these areas contains both ARG and ARB. Most antibiotics used in medicine, agriculture and the food industry, as well as resistant bacteria, end up in wastewater. Wastewater treatment plants (WWTPs) therefore could provide a comprehensive overview of ARG abundance, diversity and genomic backgrounds in particular region^3^. Moreover, wastewater and WWTPs are places where ARGs and ARB are particularly abundant and are often considered “hotspots” for the formation of strains with multiple resistance and one of the main sources of the spread of AMR in the environment^4^.

Despite numerous studies on the role of WWTPs in resistome diversity and dissemination, each new study is, in terms of time and geography, unique, as many urban areas and countries have not yet been studied. In addition, some studies are dedicated to explore only one component of the wastewater treatment system, such as wastewater, activated sludge or treated effluent, and there is a lack of research that would give a comprehensive view of the diversity and change in the composition of the resistome at different stages of water cleaning, from wastewater to treated effluent, released into the environment.

Usually, wastewater treatment in large facilities takes place in three stages. The first stage includes physical methods of water cleaning, the second stage is microbiological treatment in bioreactors with activated sludge (AS), and the third stage is the final treatment of water and its disinfection. At the second stage, than could be performed using several technologies, microorganisms of AS are used to remove organic matter, ammonium, and (in more complex processes) phosphorus^5^. At this stage, the removal of microorganisms present in the wastewater, including ARB, occurs due to their adsorption on AS particles, which are removed along with excess AS. The efficiency of this process differs for various bacteria and depends on the purification technology used. Therefore, purification technologies directly affect the removal of particular ARGs and ARB, however, this issue was poorly studied^6^.

ARGs representing all known resistance mechanisms have been found in WWTP environments^7^. ARGs for beta-lactams, macrolides, quinolones, tetracyclines, sulfonamides, trimethoprim, and multidrug efflux pump genes have been found in the incoming wastewater, AS, and treated effluent in various countries^7,8^. Recently, Munk and coauthors (2022) using metagenomics methods characterized resistomes of 757 urban wastewater samples from 243 cities in 101 countries covering 7 major geographical regions. They reported regional patterns in wastewater resistomes that differed between subsets corresponding to drug classes and were partly driven by taxonomic variation^3^. Although this study did not analyzes the composition of the wastewater resistome after treatment, there are numerous evidences that the prevalence of ARB and ARG in rivers may increase downstream from the point of discharge of treated wastewater into them^9,10^. In a study of WWTPs in Germany, 123 types of clinically significant antibiotic resistance genes were found in treated wastewater discharged into water bodies^11^. An analysis of the presence of 30 ARGs at different stages of wastewater treatment at WWTPs in Northern China showed that the content of most ARGs in the treated effluent was lower compared to the influent entering the treatment, although an increase in the abundance of some ARGs and their release into the environment was also observed^12^. A metagenomic analysis of WWTP in Hong Kong revealed seasonal changes in the content of several types of ARG and its decrease in the treated effluent^13,14^. Most ARGs were reduced by more than 98% in the treated effluent compared to the wastewater entering the treatment^14^. Some other studies have also reported a decrease in ARGs after wastewater treatment^15–17^. However, in other studies, no changes in the ARG content or even an increase were observed^17–19^. Although there are numerous studies of resistomes in WWTP-related environments the distribution of samples was geographically biased and covered mostly North America, Western Europe, Eastern Asia (mostly China), Australasia, and few places in South America/Caribbean and Sub-Saharan Africa^3^.

In order to expand the geographical coverage and our knowledge about global resistome abundance and diversity, we analyzed resistomes of wastewater before and after treatment at large-scale WWTPs in the city of Moscow (Russia). Although Moscow WWTPs are among the largest in the world and may play an important role in the spread of antibiotic resistance, the resistomes of municipal wastewaters in Moscow have not previously been studied by modern molecular genetic methods. Previously we performed 16S rRNA gene profiling of AS microbial communities at large-scale WWTPs responsible for the treatment of municipal wastewater ion Moscow^5^. Comparison of microbial communities of AS samples from WWTPs in Moscow and worldwide revealed that Moscow samples clustered together indicating the importance of influent characteristics, related to local social and environmental factors, for wastewater microbiomes^5^. For example, due to the relatively low cost of water for household consumption, wastewater in Moscow has a relatively low content of organic matter. Apparently the presence of ARB and ARGs in communal wastewater depends on the frequency of antibiotic use and the range of drugs used. These factors differ in different countries and cities. Therefore, the characterization of the resistome and the role of Moscow WWTPs in the dispersion of ARGs is an important goal. Of particular interest is also the assessment of the impact of wastewater treatment technology on the composition of the resistome and the degree of ARG removal.

Here we present the first metagenomic overview of the composition of resistome of influent wastewater, AS and treated effluent released into the environment at two Moscow WWTPs employing different treatment technologies.

## Methods

### Characteristics of WWTPs and water chemistry

The Lyuberetskiy WWTP complex of JSC “Mosvodokanal” carry out the treatment of wastewater in the city of Moscow with a capacity of about 2 million m^3^ per day. This complex consists of several wastewater treatment units (hereafter referred to as WWTPs). They purify the same inflow wastewater but otherwise are independent installations between which there is no transfer and mixing of AS. Two WWTPs implementing different technologies for wastewater treatment were chosen as the objects of study. The first one (LOS) is operated using anaerobic/anoxic/oxic process, also known as the University of Cape Town (UCT) technology. There the sludge mixture first enters the anaerobic zone, where phosphate-accumulating microorganisms (PAO) consume easily degradable organics, then to the anoxic zone, where denitrification and accumulation of phosphates by denitrifying PAO occur, and finally to the aerobic zone, where organic matter and ammonium are oxidized while PAO accumulate large quantities of polyphosphate. The second WWTP (NLOS2) uses a simpler nitrification–denitrification technology (N-DN). In the aerobic zones organics and ammonium are oxidized, while in the anoxic zone nitrate is reduced to gaseous nitrogen. This treatment technology removes organic matter and nitrogen, but was not specially aimed to remove phosphates. The production capacity of LOS is approximately 2 times more than that of NLOS2; there are no other important differences between these WWTPs besides treatment technology.

### Sampling and chemical analysis

Wastewater and AS samples were collected in September 2022 and kindly provided by “Mosvodokanal” JSC. The temperature of water samples was about 24 °C. Samples of AS from bioreactors of two WWTPs were taken in 50 ml Falcon tubes (BD Biosciences). Wastewater samples (influent and effluents from two WWTPs) were taken in 5 L plastic bottles. The cells were collected by centrifugation at 3000 g for 20 min at 4 °C.

Wastewater quality values, namely, biochemical oxygen demand (five days incubation) (BOD_5_), chemical oxygen demand (COD), total suspended solids (TSS), sludge volume index (SVI), ammonium nitrogen (N-NH_4_), nitrate nitrogen (N-NO_3_), nitrite nitrogen (N-NO_2_) and phosphorus (P-PO_4_) in the influent and effluents of two WWTPs were measured by the specialized laboratory “MSULab” according to the Federal inspection of environmental management’s protocols for chemical analyses of water.

### DNA isolation, 16S rRNA gene sequencing and analysis

Total genomic DNA was isolated using a Power Soil DNA isolation kit (Qiagen, Germany). DNA for each sample was isolated in four parallel replicates, which were then pooled. PCR amplification of 16S rRNA gene fragments comprising the V3–V4 variable regions was performed using the universal primers 341F (5′-CCTAYG GGDBGCWSCAG) and 806R (5′-GGA CTA CNVGGG THTCTAAT)^20^. The obtained PCR fragments were bar-coded and sequenced on Illumina MiSeq (2 × 300 nt reads). Pairwise overlapping reads were merged using FLASH v.1.2.11^21^. All sequences were clustered into operational taxonomic units (OTUs) at 97% identity using the USEARCH v.11 program^22^. Low quality reads were removed prior to clustering, chimeric sequences and singletons were removed during clustering by the USEARCH algorithms. To calculate OTU abundances, all reads obtained for a given sample were mapped to OTU sequences at a 97% global identity threshold by USEARCH. The taxonomic assignment of OTUs was performed by searching against the SILVA v.138 rRNA sequence database using the VSEARCH v. 2.14.1 algorithm^23^.

The diversity indices at a 97% OTU cut-off level were calculated using USEARCH v.11^22^. To avoid sequencing depth bias, the numbers of reads for each sample were randomly sub-sampled to the size of the smallest set.

### Sequencing of metagenomic DNA, contigs assembly and binning of MAGs

Metagenomic DNA was sequenced using the Illumina HiSeq2500 platform according to the manufacturer’s instructions (Illumina Inc., San Diego, CA, USA). The sequencing of a paired-end (2 × 150 bp) NEBNext Ultra II DNA Library prep kit (NEB) generated from 145 to 257 million read pairs per sample. Adapter removal and trimming of low-quality sequences (Q < 30) were performed using Cutadapt v.3.4^24^ and Sickle v.1.33 (https://github.com/najoshi/sickle), respectively. The resulting Illumina reads were de novo assembled into contigs using SPAdes v.3.15.4 in metagenomic mode^25^.

The obtained contigs were binned into metagenome-assembled genomes (MAGs) using 3 different programs: MetaBAT v.2.2.15^26^, MaxBin v.2.2.7^27^ and CONCOCT v.1.1.0^28^. The results of the three binning programs were merged into an optimized set of MAGs using DAS Tool v.1.1.4^29^. The completeness of the MAGs and their possible contamination (redundancy) were estimated using CheckM v.1.1.3^30^ with lineage-specific marker genes. The assembled MAGs were taxonomically classified using the Genome Taxonomy Database Toolkit (GTDB-Tk) v.2.0.0^31^ and Genome Taxonomy database (GTDB)^32^.

### ARG identification

Open reading frames (ORFs) were predicted in assembled contigs using Prodigal v.2.6.3^33^. ARGs were predicted using the NCBI AMRFinderPlus v.3.11.4 (https://github.com/ncbi/amr/wiki) command line tool and its associated database^34^. The predicted protein sequences of all ORFs were analyzed in this tool with parameter “-p”.

## Results

### Efficiency of wastewater treatment

Two wastewater treatment technologies were used in the investigated WWTPs,—nitrification/denitrification at NLOS2 and more advanced anaerobic/anoxic/oxic UCT process at LOS. LOS removed more than 99.5% of organic matter (according to the BOD5 data) and more than 99.9% of ammonium while the performance of NLOS2 was poorer (Table 1). Particularly noticeable differences were observed in nitrate and nitrite concentrations in the effluents suggesting the lower efficiency of denitrification in the NLOS2. Interestingly, although the NLOS2 unit was not designed to remove phosphorus, the concentration of phosphates in the treated effluent at this WWTP is only slightly higher than at LOS. The treated influent at LOS contains fewer solids consistently with lower SVI. Overall, the technology used at LOS plant is more efficient.

**Table 1:** Main parameters of influent, effluent and activated sludge in LOS and NLOS2 wastewater treatment plants.

Sample	BOD_5_ (mg/L)	COD (mg/L)	TSS (mg/L)	SVI* (mL/g)	N-NH_4_ (mg/L)	N-NO_3_ (mg/L)	N-NO_2_ (mg/L)	P-PO_4_ (mg/L)
Influent	190 ± 27	362 ± 54	NA	–	40.1 ± 3.3	< 0.1	< 0.1	3.3 ± 0.4
LOS -Effluent	1.31 ± 0.18	58 ± 12	4.5 ± 0/1	52 ± 1	< 0.05	< 0.1	0.05 ± 0.01	0.24 ± 0.03
NLOS2 -Effluent	2.10 ± 0.29	77 ± 15	8.8 ± 0.7	75 ± 6	1.0 ± 0.1	5.3 ± 0,7	0.50 ± 0.07	0.36 ± 0.05

NA, not analyzed; * data for AS.

### Microbiomes of the influent wastewater, activated sludge and treated effluent

The 16S rRNA gene profiling of microbial communities revealed 1013 species-level OTUs (97% identity) in the influent and 1.2–1.7 times more OTUs in the AS and treated effluent samples (Supplemental Table S1). The Shannon diversity indices correlated with the number of detected OTUs and increased in the series “influent” – “activated sludge” – “effluent” at each WWTP (Supplemental Table S2).

**Figure 1:**
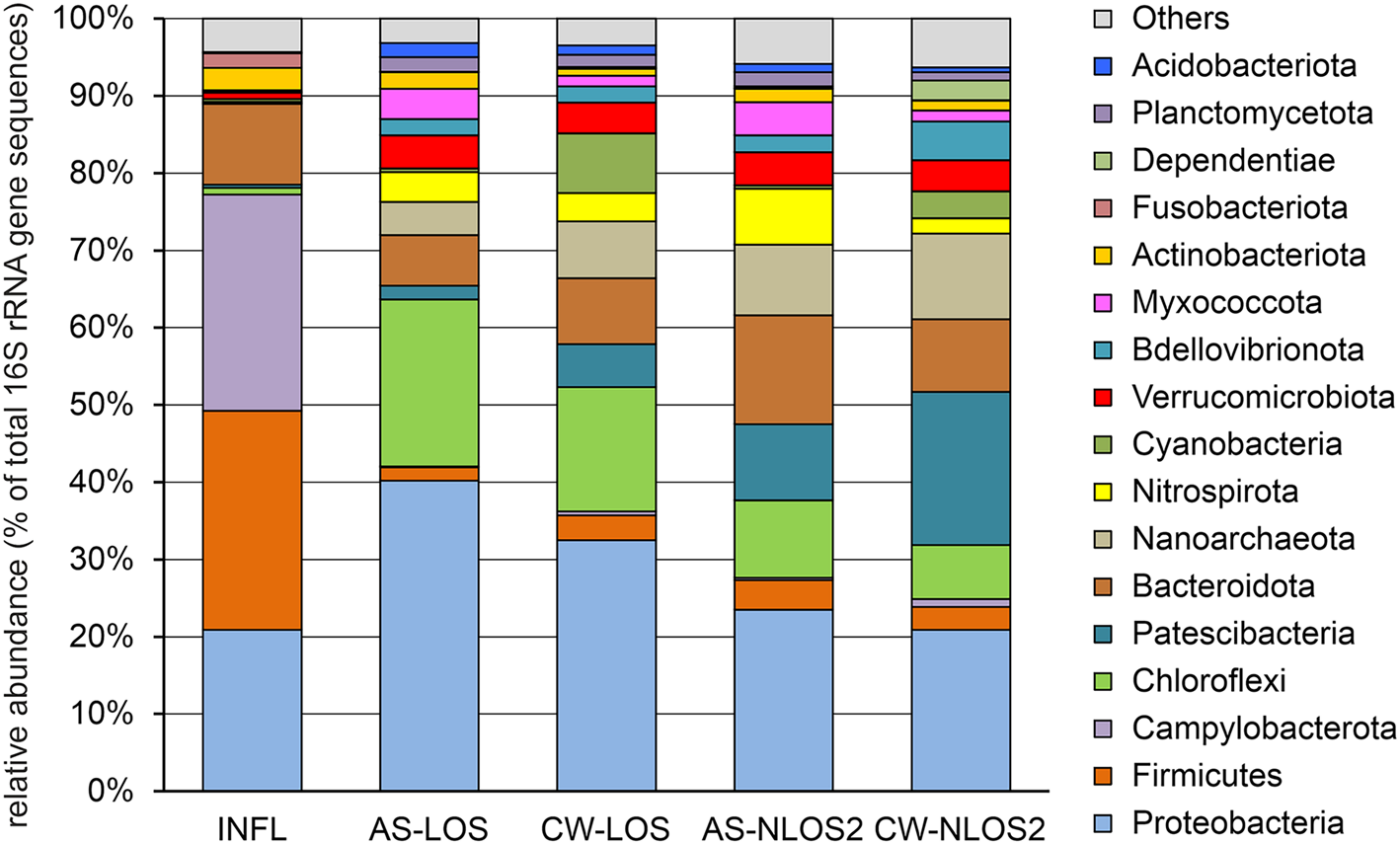
Microbial community composition in the influent, AS and treated effluent samples according to 16S rRNA gene profiling. The composition is displayed at the phylum level. INFL, influent wastewater; AS-LOS, AS at LOS plant; CW-LOS, treated effluent at LOS plant; AS-NLOS2, AS at NLOS2 plant; CW-NLOS2, treated effluent at NLOS2 plant.

Analysis of the microbiome of wastewater supplied for biological treatment showed that that the most numerous phyla in the microbial community were Firmicutes (28.4% of all 16S rRNA gene sequences), Campylobacterota (28.0%), Proteobacteria (20.9%), and Bacteroidota (10.5%) (Fig. 1). These were mainly representatives of the fecal microbiota, which are often found in wastewater. The phylum Firmicutes was dominated by *Streptococcaceae* (9.7%, mostly S*treptococcus* sp.), *Lachnospiraceae* (5.9%), *Ruminococcaceae* (3.0%), *Carnobacteriaceae* (1.7%), *Peptostreptococcaceae* (1.6%) and *Veillonellaceae* (1.4%). Most of Campylobacterota belonged to the family *Arcobacteraceae* (26.8%) of the genera *Arcobacter* (19.9%), *Pseudarcobacter* (2.5%) and uncultured lineage (4.3%), as well as by sulfur-oxidizing *Sulfurospirillum* (1.0%). Among the Proteobacteria the most abundant genera were *Acinetobacter* (7.8%)*, Aeromonas* (1.8%) and *Pseudomonas* (1.1%). Most of the identified Bacteroidota were typical fecal contaminants such as members of the genera *Bacteroides* (2.6%), *Macellibacteroides* (1.5%), *Prevotella* (1.4%), and *Cloacibacterium* (1.2%).

Activated sludge of WWTP bioreactors is a complex microbial community consisting of physiologically and phylogenetically heterogeneous groups of microorganisms involved in the removal of major contaminants from wastewater. The composition of AS microbiomes was very different from the microbiome of incoming wastewater (Fig. 1). The phyla Campylobacterota (less than 0.5%) and Firmicutes (2–4%) were much less abundant in AS microbiomes. Proteobacteria was the dominant group in the microbiomes of AS (23–40%), but its composition differed from the microbiome of influent wastewater: instead of the fecal microflora (Enterobacterales and others) the AS community harbored lineages involved in the purification processes (*Competibacteraceae*, *Rhodocyclaceae*, *Nitrosomonadaceae*, etc.). Likewise, Bacteroidota were among the most numerous phyla in AS microbiomes at both LOS (6.5%) and NLOS2 (14.1%), but instead of Bacteroidales mostly comprised Chitinophagales and Sphingobacteriales typical for AS communities. The numerous groups of AS community also included Chloroflexi (22% and 10% in LOS and NLOS2, respectively), Patescibacteria (1.8% and 9.9%), Nanoarchaeota (4.3% and 9.1%), Nitrospirota (3.9% and 7.3%), Verrucomicrobiota and Myxococcota (about 4% in both WWTPs). Bacteria that play an important role in the removal of nitrogen (*Nitrospira* and *Nitrosomonas*) and phosphorus (*Dechloromonas*), as well as glycogen-accumulating *Ca*. Competibacter, have been found in large numbers. The abundance of these functional groups is consistent with the high efficiency of nitrogen and phosphorus removal.

The main source of microorganisms in treated effluent is the AS, from which they are washed out; bacteria from the influent water may also be present in minor amounts. Therefore, as expected, the microbiome composition of treated wastewater was similar to that of activated sludge. Consistently, compositions of microbiomes of treated effluent were similar to that of AS samples. However, some differences were observed, in particular, the microbiomes of the treated effluent contained many Cyanobacteria (7.74% and 3.49% for LOS and NLOS2, respectively) which were found in minor amounts both in the influent water and in the ASs (< 0.5%). Probably, these light-dependent bacteria proliferate in the final clarifier and then can be easily washed out with the effluent.

### Diversity of resistomes

The results of metagenomic analysis of incoming wastewater revealed 544 ARGs in the assembled contigs, classified into 33 AMR gene families (Table 2 and Supplemental Table S3). Among the most numerous were classes A, C, D and metallo- beta-lactamases, rifampin ADP-ribosyltransferase, Erm 23S ribosomal RNA methyltransferase, aminoglycoside nucleotidyl-, acetyl- and phospho-transferases, the ABC-F type ribosomal protection proteins, chloramphenicol acetyltransferase, trimethoprim-resistant dihydrofolate reductase, quaternary ammonium compound efflux SMR transporters, lincosamide nucleotidyltransferases, tetracycline efflux MFS transporters and tetracycline resistance ribosomal protection proteins (Table 2). These genes may enable antibiotic inactivation (373 genes), target protection (85 genes), efflux (44 genes) and target replacement (25 genes).

**Table 2:** Resistomes of wastewater and AS samples, classified by gene families.

	Number of ARGs				
	INFL	AS- LOS	CW-LOS	AS-NLOS2	CW-NLOS2
Class A beta-lactamase	77	22	38	20	47
Class C beta-lactamase	22	2	4	3	6
Class D beta-lactamase	75	35	39	39	52
Metallo-beta-lactamase	24	37	43	43	71
Rifampin ADP-ribosyltransferase	60	33	54	63	81
Erm 23S ribosomal RNA methyltransferase	34	10	13	14	17
Aminoglycoside acetyltransferase	19	9	7	10	11
Aminoglycoside nucleotidyltransferase	22	8	9	7	13
Aminoglycoside phosphotransferase	10	5	6	6	9
ABC-F type ribosomal protection	22	4	6	3	4
Chloramphenicol acetyltransferase	20	5	5	9	5
Trimethoprim-resistant dihydrofolate reductase	19	3	4	2	2
Quaternary ammonium compound efflux SMR transporter	14	6	8	7	10
Lincosamide nucleotidyltransferase	13	4	5	5	8
Tetracycline efflux MFS transporter	12	6	6	7	6
Tetracycline resistance ribosomal protection protein	12	6	6	4	7
Others	89	26	38	24	44
Total	544	221	291	266	393

**Figure 2:**
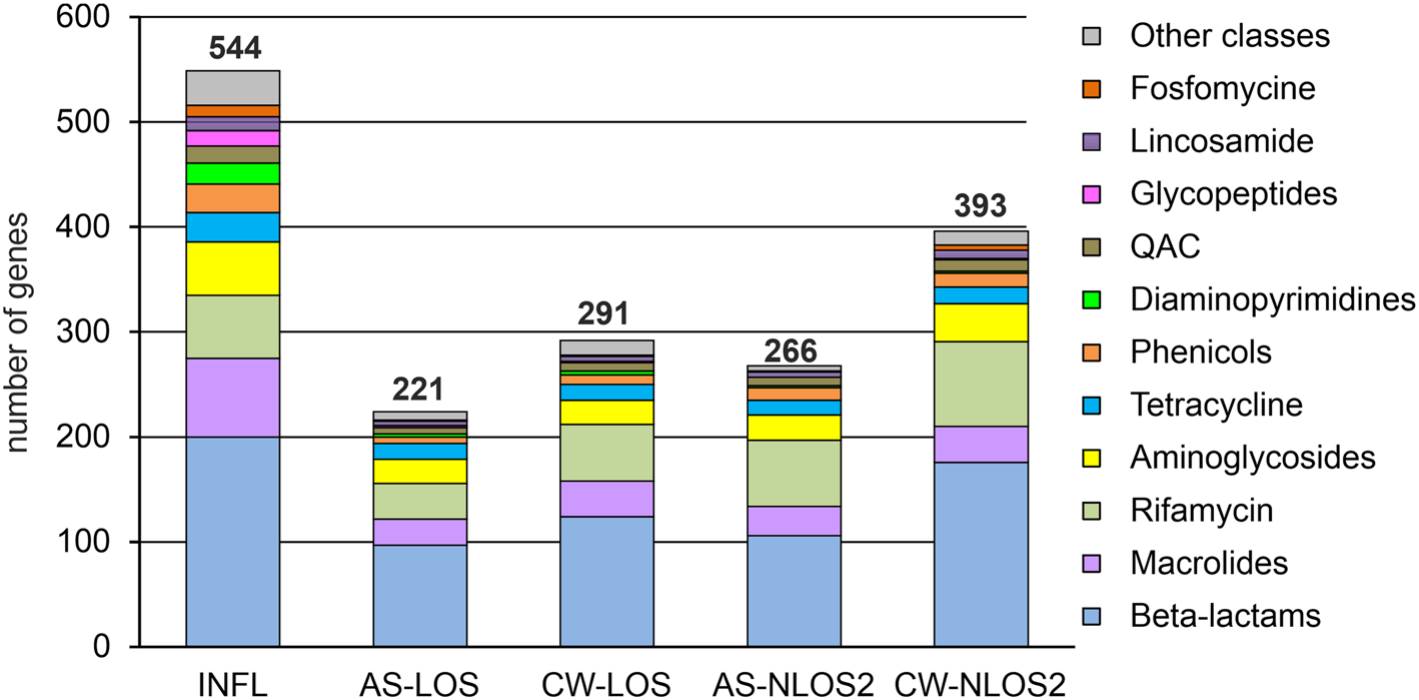
ARGs identified in wastewater and AS samples categorized by drug classes.

The abovementioned genes confer resistance to most of commonly used drugs: beta-lactams (198 genes), macrolides (74 genes), rifamycin (60 genes), aminoglycosides (51 genes), tetracycline (27 genes), phenicols (27 genes), diaminopyrimidines (19 genes), quaternary ammonium compounds (16 genes), glycopeptides (15 genes), lincosamide (13 genes), fosfomycine (12 genes) and drugs of 11 others classes (Fig. 2).

About twice less ARGs were identified in AS samples from both WWTPs. Like in the influent, beta-lactamases of classes A, D, and metallo-beta-lactamases were the most numerous, while only a few genes for class C enzymes were found (Table 2). Other families of ARGs, numerous in the influent, were also numerous in AS microbiomes. A notable difference between the resistomes of the AS samples is the greater number of rifampin-ADP-ribosyltransferase genes (*arr*) in NLOS2 compared to LOS (63 vs 33). The largest number of *arr* genes was assigned to Bacteroidota, and the lower relative abundance of this phylum in AS at LOS likely explains these differences. Like in the wastewater, resistance to beta-lactams, macrolides, rifamycin, aminoglycosides, and tetracyclines was the most common (Fig. 2). On the contrary, genes for some drug classes were underrepresented in AS resistomes, especially for diaminopyrimidines (3 and 2 genes for LOS and NLOS2, respectively) and glycopeptide antibiotics (2 and 0 genes).

The results of metagenomic analysis of treated effluent showed that the diversity of these resistomes was only slightly higher than that of the corresponding AS samples. This result was expected since the main source of microorganisms in the effluent is activated sludge, from which they are partially washed. However, resistomes of treated effluent at both WWTPs contains about twice more class A beta-lactamase genes than AS samples suggesting less efficient absorption of their host bacteria at AS particles (Table 2).

### Quantitative analysis of antibiotic resistance genes of WWTP

The results described above provide information on the diversity of resistance genes, but not on their abundance in the metagenomes, which depends on the abundance of corresponding bacterial hosts. To quantify the shares of individual ARGs in the metagenome and resistome, the amounts of metagenomic reads mapped to the corresponding ARGs in contigs were determined. In total, the resistome accounted for about 0.05% of the metagenome of wastewater supplied for treatment, while the shares of resistomes in the metagenomes of AS and treated effluent samples were 0.02% and 0.014% at the LOS and NLOS2 WWTPs, respectively.

**Figure 3:**
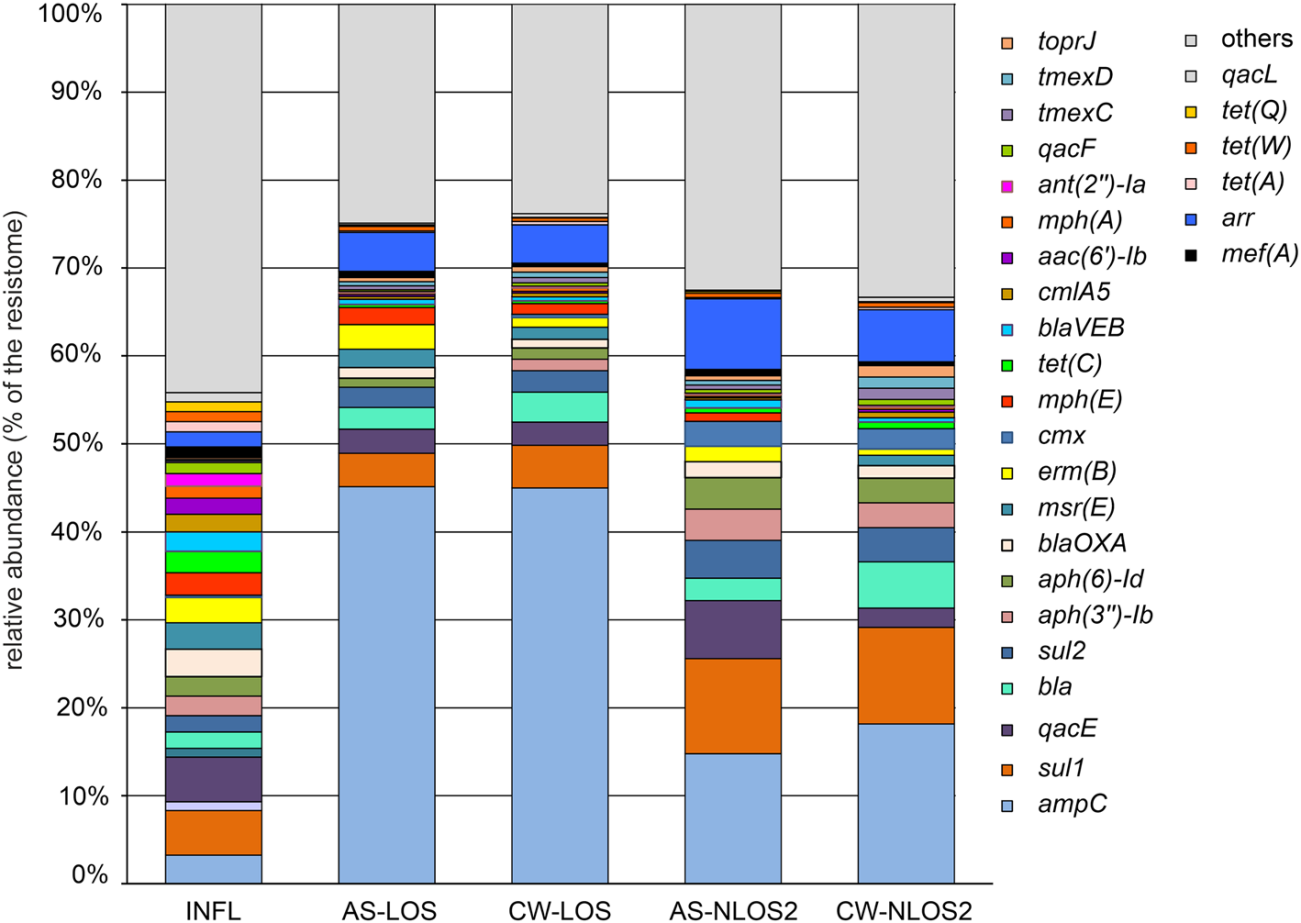
The relative abundancies of particular ARGs in the resistomes. Only ARGs with shares greater than 1% in at least one sample are shown, all other ARGs are shown as “others”.

Quantitative analysis of the content of individual ARGs in metagenomes showed that the structure of the influent resistome was very different from that of AS and treated effluent. The relative content of ARGs accounting for more than 1% in at least one analyzed resistome is shown in Fig. 3. The LOS and NLOS2 WWTPs differed significantly from each other, and the differences between the AS and effluent resistomes at each WWTP were much less pronounced.

The resistome of the influent was not only the most diverse, but also the most even in composition. The shares of none of the ARGs exceeded 5% of the resistome, and the 23 most common ARGs accounted for a half of the resistome. The most abundant ten ARGs were *qacE, sul1, ampC, blaOXA, msr(E), erm(B), mph(E), tet(C), aph(3'')-Ib* and *aph(6)-Id,* conferring resistance to antiseptics, sulfonamides, beta-lactams, macrolides, aminoglycosides (streptomycin), and tetracyclines.

AS and treated effluent at LOS plant was strongly dominated by a single AGR type, class C beta-lactamase *ampC*, accounting for about 45% of their resistomes. This gene was also the most abundant one in the resistomes of AS and effluent at NLOS2 (14.8% and 18.2%, respectively). Apparently it originates from the influent wastewater supplied for treatment where its share in the resistome was 3.2%. AmpC β-lactamases are considered clinically important cephalosporinases encoded on the chromosomes and plasmids of various bacteria (especially *Enterobacteriaceae*), where they mediate resistance to cephalothin, cefazolin, cefoxitin and most penicillins^35^. Close homologues of this gene, with a nucleotide sequence identity of 99.8–100%, have been found in plasmids and chromosomes of various Proteobacteria (*Thauera, Sphingobium, Aeromonas* etc.). Since in all samples *ampC* was found in short contigs with very high coverage, it is likely widespread in the genomes of various bacteria in different genetic contexts.

The second most abundant ARG in the resistomes of AS samples was sulfonamide-resistant dihydropteroate synthase (*sul1*). It accounted for 4–5% of AS and treated effluent resistomes in LOS and for about 11% in NLOS2, while its share in the influent water resistome was about 5%. The *sul1* gene is usually found in class 1 integrons being linked to other resistance genes, including *qacE*^36^. Consistently, *sul1* and *qacE* were found in one contig assembled for the influent water samples and assigned to Gammaproteobacteria. Another sulfonamide-resistance gene, *sul2*, was also numerous, accounting for about 2% of the resistomes in the influent and LOS samples, and for about 4% in the AS and water treated at NLOS2.

Since ARGs entering the activated sludge and then into the treated effluent originate mostly from wastewater supplied for treatment, the absolute majority of ARGs present in the influent in significant amounts (more than 0.2% resistome) in were also found in AS and effluent samples. The only exception macrolide 2′-phosphotransferase gene *mph(B)* accounting for 0.51% in the influent resistome. Likewise, all ARGs accounting for more than 0.2% of resistomes in the treated effluent were present also in the influent.

### Potential multidrug resistant strains

One of the most important public health problems is the spread of multidrug resistant pathogens (MDR), which refers to resistance to at least one agent in three or more chemical classes of antibiotic (e.g. a beta-lactam, an aminoglycoside, a macrolide)^37^. Such strains can arrive with wastewater entering the treatment, and also form in AS communities. AS are dense and highly competitive microbial communities, which, along with the presence of sublethal concentrations of antibiotics and other toxicants in wastewater, creates ideal conditions not only for the selection of resistant strains, but also for the formation of multiple resistance through horizontal gene transfer^4^. To identify MDR bacteria, we binned metagenomic contigs into metagenome-assembled genomes (MAGs) and looked for MAGs comprising several ARGs. Only MAGs with more than 70% completeness and less than 15% contamination were selected for analysis: 117, 56, 72, 94 and 121 for influent, AS of LOS, effluent of LOS, AS of NLOS2 and effluent of NLOS2, respectively. Five MAGs of MDR bacteria were identified in the metagenome of the influent, one—in AS of LOS, two—in the LOS effluent and one in the NLOS2 effluent (Table 3). These MAGs were assigned to unclassified genus-level lineages of *Ruminococcaceae* and *Cyclobacteriaceae, Phocaeicola vulgatus, Streptococcus parasuis, Ancrocorticia* sp., *Enterococcus* sp., *Bacillus cereus* and *Undibacterium* sp.

**Table 3:** MAGs comprising multiple ARGs.

Sample	MAG taxonomy according to GTDB	Bin completeness/contamination (%)	ARGs	Drug class
Influent	p__Firmicutes_A; c__Clostridia; o__Oscillospirales; f__Ruminococcaceae	81.43/0.22	*vanR*	glycopeptide
			*catP*	phenicol
			*abc-f*	macrolide
	p__Bacteroidota; c__Bacteroidia; o__Bacteroidales; f__Bacteroidaceae; g__Phocaeicola; s__Phocaeicola vulgatus	88.2/1.5	*bla*	beta-lactam
			*lnu(AN2)*	lincosamide
			*mef(En2)*	macrolide
			*bla*	beta-lactam
	p__Firmicutes; c__Bacilli; o__Lactobacillales; f__Streptococcaceae; g__Streptococcus; s__Streptococcus parasuis	86.0/9.0	*tet(Q)*	tetracycline
			*msr(D)*	macrolide
			*mef(A)*	macrolide
			*mph(B)*	macrolide
			*tet(M)*	tetracycline
			*bla*	beta-lactam
			*tet(32)*	tetracycline
			*tet*	tetracycline
	p__Actinobacteriota; c__Actinomycetia; o__Actinomycetales; f__Actinomycetaceae; g__Ancrocorticia	91.0/4.6	*aac(3)-IId*	aminoglycoside
			*catA*	phenicol
			*erm*	macrolide
	p__Firmicutes; c__Bacilli; o__Lactobacillales; f__Enterococcaceae; g__Enterococcus_F	98.7/0.3	*catA*	phenicol
			*arr*	rifamycin
			*abc-f*	macrolide
			*lsa*	lincosamide/streptogramin
			*abc-f*	macrolide
AS of LOS	p__Firmicutes; c__Bacilli; o__Lactobacillales; f__Streptococcaceae; g__Streptococcus	81.6/9.5	*aadE*	aminoglycoside
			*lnu(C)*	lincosamide
			*tet*	tetracycline
			*blaB*	beta-lactam
Effluent of LOS	p__Bacteroidota; c__Bacteroidia; o__Cytophagales; f__Cyclobacteriaceae; g__ELB16-189; s__ELB16-189 sp002352225	77.5/15.0	*bla*	beta-lactam
			*blaB1*	beta-lactam
			*lnu(D)*	lincosamide
			*vat*	streptogramin
	p__Firmicutes; c__Bacilli; o__Bacillales; f__Bacillaceae_G; g__Bacillus_A; s__Bacillus_A cereus	91.1/10.0	*abc-f*	macrolide
			*bla2*	beta-lactam
			*bla*	beta-lactam
			*fosB*	fosfomycin
			*bla1*	beta-lactam
			*vat*	streptogramin
			*abc-f*	macrolide
Effluent of NLOS2	p__Proteobacteria; c__Gammaproteobacteria; o__Burkholderiales; f__Burkholderiaceae; g__Undibacterium	71.4/2.3	*fos*	fosfomycin
			*aph(3')*	aminoglycoside
			*bla*	beta-lactam
			*blaOXA*	beta-lactam

## Disscussion

We characterized the composition of microbial communities and the resistomes of influent wastewater, activated sludge and treated effluent from two WWTPs in city of Moscow, where various biological water treatment technologies are used. Among the predominant bacteria in the influent wastewater we found mainly fecal contaminants of the genera *Collinsella*, *Bacteroides*, *Prevotella*, *Arcobacter*, *Arcobacteraceae*, *Blautia*, *Faecalibacterium, Streptococcus*, *Acinetobacter*, *Aeromonas* and *Veillonella*^38–43^. Previously, we performed 16S rRNA gene profiling of wastewater before and after treatment at one WWTP (LOS) and revealed that all abovementioned potential pathogens were efficiently removed and their relative abundance in the water microbiome decreased by 50‒100 times^44^. Similar pattern of removal of potential pathogenic bacteria was observed here for NLOS2 where another water treatment technology is used.

An important indicator of the dissemination of ARG is the proportion of the resistome in the entire metagenome before and after wastewater treatment. In the influent, the resistome accounted for about 0.05% of the metagenome, which corresponds to approximately two ARGs per bacterial genome. Approximately the same values are typical for most countries^3^. After treatment, the fraction of the resistome in the wastewater metagenomes decreases, but, surprisingly, only by 2–4 times. However, since the total concentration of microorganisms in treated effluent is approximately two orders of magnitude lower than in raw wastewater, it is likely that the total abundance of ARGs in the treated effluent is significantly reduced.

Apparently, fecal contaminants effectively removed during treatment are not the only carriers of ARG in wastewater, which are also found in bacteria characteristic of activated sludge and thus appearing in the effluents. Unfortunately, due to the high diversity of microbiomes and the tendency of ARG to be present in multiple copies in different genomic environments, most of the contigs containing ARG turned out to be short, which did not allow to define their taxonomic affiliation.

The resistome of influent water includes 26 ARGs, the share of which is more than 1%. Among of them the prevalence of *ampC, aadA, qacE, bla, qacF* and *qacL* is specific for Moscow WWTPs, since these genes were not among the 50 most common ARGs according to the results of a worldwide analysis of wastewater resistomes in large cities^3^. Different ARGs were most “evenly” represented in the influent wastewater while in the AS and treated effluent, a clear selection of particular types of ARGs was observed, which obviously reflects a change in the composition of microbiomes. A vivid example is the increase in the proportion of *ampC* in the resistomes, especially at LOS.

**Figure 4:**
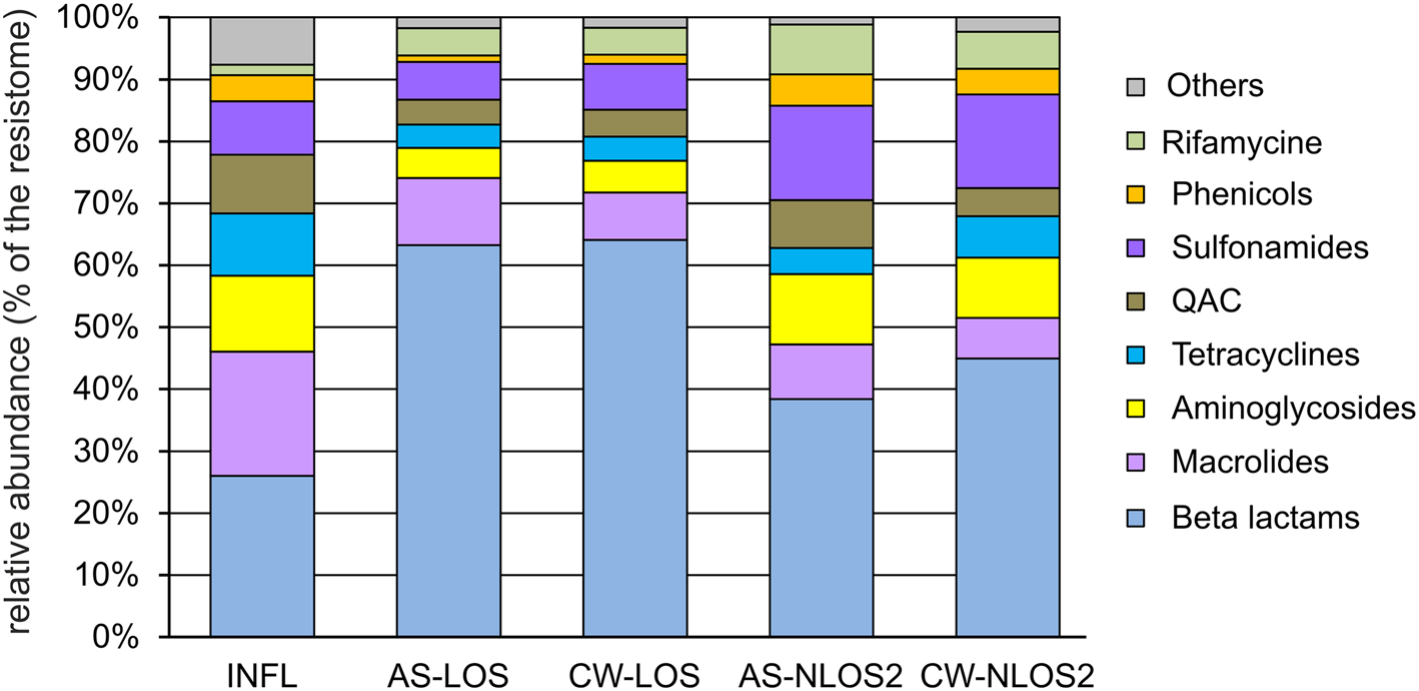
The relative abundancies of ARGs in the resistomes categorized by drug classes.

The discovered ARGs can confer resistance to most classes of antibiotics and among the resistomes of the studied WWTPs in the city of Moscow, genes conferring resistance to beta-lactam antibiotics were the most common, they accounted for about 26% of the resistome in the water supplied for treatment (Fig. 4). Similar values have been observed for wastewater in some other countries, particularly in Eastern Europe and Brazil, where 20 to 25% of reads were assigned to ARGs conferring resistance to beta-lactams^3^. According to data for 2021, beta lactams accounted for about 40% of the total antibiotic consumption in Russia in the medical sector^45^.

Like in most wastewater resistomes in different countries, ARGs conferring resistance to macrolides, aminoglycosides and tetracycline were also among the most abundant in wastewater from Moscow (Fig. 4). Resistance to macrolides, rather than beta-lactams, was most common in wastewater from most countries in Europe and North America, while in Moscow ARGs to macrolide were the second most common. Macrolides and tetracyclines are also widely used in medicine in Russia (20% and 5% of total antibiotic consumption in 2021, respectively). On the contrary, medical consumption of aminoglycosides in Russia is rather low (< 1% of the total), therefore, the high abundance of relevant ARGs was unexpected. The opposite pattern was observed for quinolones, which make up about 22% of the antibiotics used in medicine, but their ARGs accounted for only about 1% of the resistome. However the main mechanisms of resistance to quinolones, mutations in the target enzymes, DNA gyrase and DNA topoisomerase IV, and increased drug efflux^46^, were not addressed in our study.

A peculiar feature of Moscow wastewater resistome was the high content of resistance genes to sulfonamides (about 9%), which were not among the major genes in wastewater resistomes worldwide^3^. Sulfonamides are synthetic antimicrobial agents that currently have limited use in the human medicine, alone or mainly in combination with trimethoprim (a dihydrofolate reductase inhibitor), in the treatment of uncomplicated respiratory, urinary tract and chlamydia infections^7,47^. Different sulfonamide ARGs (*sul1, sul2* and *sul3*) were detected in the wastewater in the some countries, including Denmark, Canada, Spain and China, applying culture dependent, independent and qPCR methods^7^. The opposite picture was observed for streptogramin resistance genes, which were among the ARGs in the majority of resistomes worldwide, but in Moscow wastewater they accounted for less than 1%. This is probably due to the limited use of this drug in Russia.

Another distinguishing feature of the resistome of wastewater in Moscow is the high content of ARGs conferring resistance to quaternary ammonium compounds (QAC), about 9%. It can be explained by the frequent use of these antiseptics in medicine. QACs are active ingredients in more than 200 disinfectants currently recommended for inactivation the SARS-CoV-2 (COVID-19) virus^48^. A recent study showed that the number of QACs used to inactivate the virus in public facilities, hospitals and households increased during the COVID-19 pandemic^49^. Indeed, the results of a study dedicated to the study of wastewater resistome worldwide^3^ did not reveal the presence of QAC ARGs in the wastewater, since the samples for this study were collected before the pandemic.

An important issue is the extent to which different water treatment technologies remove ARGs. The effective removal of ARG was primary due to a decrease in the concentration of microorganisms in treated effluent, since the share of resistome in the metagenome after treatment decreased by only 2.6 –3.7 times and the NLOS2 plant appeared to be more effective in this respect. However, compared to LOS, treated effluent at NLOS2 contains approximately twice as much suspended solids, probably due to poorer settling characteristics of the sludge indicated by the higher SVI. Therefore, the overall efficiency of removing ARGs from wastewater at two WWTPs may be similar.

Considering the relative abundances of ARGs in the resistomes, genes conferring resistance to macrolides and tetracyclines were removed more efficiently than beta lactamases, especially *ampC*, and rifampin ADP-ribosyltransferase genes. The low efficiency of removal of the *ampC* gene and the increase in its abundance in the resistome after wastewater treatment were previously reported for WWTPs in Germany^50^. Efficient removal of ARGs to macrolides (*ermB, ermF, mph(A), mef(A)*) and tetracyclines (*tet(A), tet(C), tet(Q), tet(W)*) has been reported in a number of studies worldwide^51^. ARGs enabling resistance to sulfonamides, tetracyclines and chloramphenicol were more efficiently removed at LOS than at NLOS2, while the opposite was observed for beta lactamases (Fig. 4). The later became the most abundant class of ARGs in the treated effluent.

Metagenomic analysis not only identified resistance genes, but also revealed probable MDR strains based on the analysis of assembled MAGs. We identified 9 such strains in both influent, AS and treated effluent. The real number of MDR strains is probably higher, since only a small fraction of all metagenomic contigs was included in the assembled high quality MAGs.

*Phocaeicola vulgatus*, (formerly *Bacteroides vulgatus*), is a mutualistic anaerobic bacteria commonly found in the human gut microbiome and frequently involved in human infections. The results of whole genome analysis showed presence of *blaTEM-1* and *blaCMY-2* ARGs, which confers resistant to beta-lactams^52,53^. *P. vulgatus* was also identified as potential host for the transmission of tetracycline ARGs^54^. *Streptococcus parasuis* is an important zoonotic pathogen that causes primarily meningitis, sepsis, endocarditis, arthritis, and pneumonia in both pigs and humans^55^. A variety of MDR strains of this bacterium have been described. For instance, *S. parasuis* strain H35 was isolated from a lung sample of a pig in China; several ARGs, including *optrA*, *catQ*, *erm(B), lsa(E), msr(D), mef(A), mdt(A), tet(M), lnu(B), aadE* and two copies of *aacA-aphD*, were found in the chromosome and *cfr(D)* was detected on plasmid pH35-cfrD^56^. MDR strain of *Bacillus cereus* was identified in the effluent water microbiome. This bacterium is known as human pathogen and a common cause of food poisoning with toxin-producing property^57^. *Bacillus cereus* was isolated from drinking water treatment plant in China and antimicrobial susceptibility testing revealed that it was resistant to cefoxitin, penicillin tetracycline^58^, macrolide-lincosamide-streptogramin (MLSB), aminoglycoside and tetracycline antibiotics^59^. Assembled MAG *B.cereus* from effluent water contained ARGs conferring to macrolides, beta-lactams, fosfomycin and streptogramin and may be considered as MDR strain. Genomes of members of the genera *Streptococcus* (AS of LOS) and *Enterococcus* (influent), not identified at the species level, were found to contain multiple ARGs. Most of species of these genera are opportunistic and true pathogens known for their drug resistance^60,61^. One MAG from the influent water metagenome was assigned to uncultured lineage of the family *Ruminococcaceae.* Members of this family are typical non-pathogenic gut inhabitants, although genomes of some strains could harbor ARGs^62^.

Three MAGs retrieved from influent wastewater microbiome (*Ancrocorticia*) and treated effluent water (*Cyclobacteriaceae* and *Undibacterium*) were found to contain several ARGs. However, we found no evidences about pathogenic and MDR strains in these taxa. It is possible that these environmental bacteria acquired ARGs via horizontal gene from outside their lineages. WWTPs are an ideal environment for horizontal gene transfer (HGT), since when bacteria are exposed to strong selective pressures, such as the presence of antimicrobials, the horizontal acquisition of ARGs enables genetic diversification and create the potential for rapid gains in fitness^63^.

## Conclusions

- Metagenome sequencing of the raw wastewater, activated sludge and treated wastewater at two large WWTPs of the Moscow city revealed several hundreds of ARGs that could confer resistance to most commonly used classes of antibiotics.
- Resistome accounted for about 0.05% of the wastewater metagenome and after wastewater treatment its share decreased by 3–4 times.
- The resistomes were dominated by ARGs encoding resistance to beta-lactams, macrolides, aminoglycosides, tetracycline, QAC, and sulfonamides. A peculiar feature of Moscow wastewater resistome was the high content of ARGs to sulfonamides and limited occurrence of resistance to streptogramins.
- ARGs for macrolides and tetracyclines were removed more efficiently than ARGs for beta-lactamases.
- A comparison of wastewater resistomes from Moscow and around the world suggested that the abundance and content of ARG in wastewater depend on social, medical, and environmental factors.

### Supplementary Information

Supplementary Tables.

## Data Availability

The raw data generated from 16S rRNA gene sequencing and metagenome sequencing have been deposited in the NCBI Sequence Read Archive (SRA) and are available via the BioProject PRJNA945245.

## References

[1] Cassini A (2019). Attributable deaths and disability-adjusted life-years caused by infections with antibiotic-resistant bacteria in the EU and the European Economic Area in 2015: A population-level modelling analysis. Lancet Infect. Dis..

[2] Thorpe KE, Joski P, Johnston KJ (2018). Antibiotic-resistant infection treatment costs. antibiotic-resistant infection treatment costs have doubled since 2002, now exceeding $2 billion annually. Health Aff..

[3] Munk P (2022). Genomic analysis of sewage from 101 countries reveals global landscape of antimicrobial resistance. Nat. Commun..

[4] Rizzo L (2013). Urban wastewater treatment plants as hotspots for antibiotic resistant bacteria and genes spread into the environment: a review. Sci. Total Environ..

[5] Begmatov S (2022). The structure of microbial communities of activated sludge of large-scale wastewater treatment plants in the city of Moscow. Sci. Rep..

[6] Mosaka TBM, Unuofin JO, Daramola MO, Tizaoui C, Iwarere SA (2022). Inactivation of antibiotic-resistant bacteria and antibiotic-resistance genes in wastewater streams: Current challenges and future perspectives. Front. Microbiol..

[7] Pazda M, Kumirska J, Stepnowski P, Mulkiewicz E (2019). Antibiotic resistance genes identified in wastewater treatment plant systems—A review. Sci. Total Environ..

[8] Karkman A, Do TT, Walsh F, Virta MPJ (2018). Antibiotic-resistance genes in waste water. Trends Microbiol..

[9] Amos GC, Hawkey PM, Gaze WH, Wellington EM (2014). Waste water effluent contributes to the dissemination of CTX-M-15 in the natural environment. J. Antimicrob Chemother..

[10] Marti E, Jofre J, Balcazar JL (2013). Prevalence of antibiotic resistance genes and bacterial community composition in a river influenced by a wastewater treatment plant. PLoS ONE.

[11] Szczepanowski R (2009). Detection of 140 clinically relevant antibiotic-resistance genes in the plasmid metagenome of wastewater treatment plant bacteria showing reduced susceptibility to selected antibiotics. Microbiology.

[12] Mao D (2015). Prevalence and proliferation of antibiotic resistance genes in two municipal wastewater treatment plants. Water Res..

[13] Yang Y, Li B, Ju F, Zhang T (2013). Exploring variation of antibiotic resistance genes in activated sludge over a four-year period through a metagenomic approach. Environ. Sci. Technol..

[14] Yang Y, Li B, Zou S, Fang HH, Zhang T (2014). Fate of antibiotic resistance genes in sewage treatment plant revealed by metagenomic approach. Water Res..

[15] Bengtsson-Palme J (2016). Elucidating selection processes for antibiotic resistance in sewage treatment plants using metagenomics. Sci. Total Environ..

[16] Karkman A (2016). High-throughput quantification of antibiotic resistance genes from an urban wastewater treatment plant. FEMS Microbiol Ecol..

[17] Laht M (2014). Abundances of tetracycline, sulphonamide and beta-lactam antibiotic resistance genes in conventional wastewater treatment plants (WWTPs) with different waste load. PLoS ONE.

[18] Auerbach EA, Seyfried EE, McMahon KD (2007). Tetracycline resistance genes in activated sludge wastewater treatment plants. Water Res..

[19] Harris SJ, Cormican M, Cummins E (2012). Antimicrobial residues and antimicrobial-resistant bacteria: Impact on the microbial environment and risk to human health—a review. Human Ecol. Risk Assess. Int. J..

[20] Frey B (2016). Microbial diversity in European alpine permafrost and active layers. FEMS Microbiol. Ecol..

[21] Magoc T, Salzberg S (2011). FLASH: Fast length adjustment of short reads to improve genome assemblies. Bioinformatics.

[22] Edgar RC (2010). Search and clustering orders of magnitude faster than BLAST. Bioinformatics.

[23] Rognes T, Flouri T, Nichols B, Quince C, Mahé F (2016). VSEARCH: A versatile open source tool for metagenomics. PeerJ.

[24] Martin M (2011). Cutadapt removes adapter sequences from high-throughput sequencing reads. EMBnet.journal.

[25] Nurk S, Meleshko D, Korobeynikov A, Pevzner PA (2017). metaSPAdes: A new versatile metagenomic assembler. Genome Res..

[26] Kang DD (2019). MetaBAT 2: an adaptive binning algorithm for robust and efficient genome reconstruction from metagenome assemblies. PeerJ..

[27] Wu YW, Simmons BA, Singer SW (2016). MaxBin 2.0: An automated binning algorithm to recover genomes from multiple metagenomic datasets. Bioinformatics..

[28] Alneberg J (2014). Binning metagenomic contigs by coverage and composition. Nat. Methods.

[29] Sieber CMK (2018). Recovery of genomes from metagenomes via a dereplication, aggregation and scoring strategy. Nat. Microbiol..

[30] Parks DH, Imelfort M, Skennerton CT, Hugenholtz P, Tyson GW (2015). CheckM: assessing the quality of microbial genomes recovered from isolates, single cells, and metagenomes. Genome Res..

[31] Chaumeil PA, Hugenholtz P, Parks DH (2022). GTDB-Tk v2: memory friendly classification with the genome taxonomy database. Bioinformatics.

[32] Parks DH (2018). A standardized bacterial taxonomy based on genome phylogeny substantially revises the tree of life. Nat. Biotechnol..

[33] Hyatt D (2010). Prodigal: prokaryotic gene recognition and translation initiation site identification. BMC Bioinform..

[34] Feldgarden M (2021). AMRFinderPlus and the reference gene catalog facilitate examination of the genomic links among antimicrobial resistance, stress response, and virulence. Sci. Rep..

[35] Jacoby GA (2009). AmpC beta-lactamases. Clin. Microbiol. Rev..

[36] Antunes P, Machado J, Sousa JC, Peixe L (2005). Dissemination of sulfonamide resistance genes (sul1, sul2, and sul3) in Portuguese *Salmonella enterica* strains and relation with integrons. Antimicrob. Agents Chemother..

[37] Magiorakos AP (2012). Multidrug-resistant, extensively drug-resistant and pandrug-resistant bacteria: An international expert proposal for interim standard definitions for acquired resistance. Clin. Microbiol. Infect..

[38] Azcarate-Peril MA (2017). Impact of short-chain galactooligosaccharides on the gut microbiome of lactose-intolerant individuals. Proc. Natl. Acad. Sci. USA.

[39] Koskey AM (2014). *Blautia* and *Prevotella* sequences distinguish human and animal fecal pollution in Brazil surface waters. Environ. Microbiol. Rep..

[40] Altwegg M, Geiss HK (1989). *Aeromonas* as a human pathogen. Crit. Rev. Microbiol..

[41] Wexler AG, Goodman AL (2017). An insider's perspective: *Bacteroides* as a window into the microbiome. Nat. Microbiol..

[42] Collado L, Figueras MJ (2011). Taxonomy, epidemiology, and clinical relevance of the genus *Arcobacter*. Clin. Microbiol. Rev..

[43] Antunes LC, Visca P, Towner KJ (2014). *Acinetobacter baumannii*: evolution of a global pathogen. Pathog. Dis..

[44] Begmatov SA, Dorofeev AG, Pimenov NV, Mardanov AV, Ravin NV (2023). High efficiency of removal of pathogenic microorganisms at wastewater treatment plants in the city of Moscow. Microbiology.

[45] Zakharenkov IA, Rachina SA, Kozlov RS, Belkova YuA (2022). Consumption of systemic antibiotics in the Russian Federation in 2017–2021. Clin. Microbiol. Antimicrob. Chemother..

[46] Hooper DC, Jacoby GA (2015). Mechanisms of drug resistance: quinolone resistance. Ann. N.Y. Acad. Sci..

[47] Littlefield BA, Gurpide E, Markiewicz L, McKinley B, Hochberg RB (1990). A simple and sensitive microtiter plate estrogen bioassay based on stimulation of alkaline phosphatase in Ishikawa cells: estrogenic action of Δ5 adrenal steroids. Endocrinology.

[48] Hora PI, Pati SG, McNamara PJ, Arnold WA (2020). Increased use of quaternary ammonium compounds during the SARS-CoV-2 pandemic and beyond: consideration of environmental implications. Environ. Sci. Technol. Lett..

[49] Liu C (2023). Low concentration quaternary ammonium compounds promoted antibiotic resistance gene transfer via plasmid conjugation. Sci. Total. Environ..

[50] Alexander J, Bollmann A, Seitz W, Schwartz T (2015). Microbiological characterization of aquatic microbiomes targeting taxonomical marker genes and antibiotic resistance genes of opportunistic bacteria. Sci. Total. Environ..

[51] Uluseker C (2021). A Review on Occurrence and spread of antibiotic resistance in wastewaters and in wastewater treatment plants: mechanisms and perspectives. Front. Microbiol..

[52] Vázquez-López R (2021). The beta-lactam resistome expressed by aerobic and anaerobic bacteria isolated from human feces of healthy donors. Pharmaceuticals.

[53] Vishwanath S, Shenoy PA, Chawla K (2019). Antimicrobial resistance profile and *nim* gene detection among *Bacteroides fragilis* group isolates in a university hospital in South India. J. Glob. Infect. Dis..

[54] Qin R, Yu QG, Liu ZY, Wang H (2023). Co-occurrence of tetracycline antibiotic resistance genes and microbial communities in plateau wetlands under the influence of human activities. Huan Jing Ke Xue.

[55] Wang J (2021). Investigation of the genomic and pathogenic features of the potentially zoonotic *Streptococcus parasuis*. Pathogens.

[56] Zhu Y (2021). Identification of a *Streptococcus parasuis* isolate co-harbouring the oxazolidinone resistance genes *cfr(D)* and *optrA*. J. Antimicrob. Chemother..

[57] Enosi TD, Mathur A, Ngo C, Man SM (2021). *Bacillus cereus*: Epidemiology, virulence factors, and host-pathogen interactions. Trends Microbiol..

[58] Gu Q (2022). Characteristics of antibiotic resistance genes and antibiotic-resistant bacteria in full-scale drinking water treatment system using metagenomics and culturing. Front. Microbiol..

[59] Han Z (2020). Antibiotic resistomes in drinking water sources across a large geographical scale: Multiple drivers and co-occurrence with opportunistic bacterial pathogens. Water Res..

[60] Fiore E, Van TD, Gilmore MS (2019). Pathogenicity of *Enterococci*. Microbiol. Spectr..

[61] Patterson MJ, Baron S (1996). Streptococcus. Medical Microbiology.

[62] Abdugheni R (2023). Comparative genomics reveals extensive intra-species genetic divergence of the prevalent gut commensal *Ruminococcus gnavus*. Microb. Genom..

[63] Lerminiaux NA, Cameron ADS (2019). Horizontal transfer of antibiotic resistance genes in clinical environments. Can. J. Microbiol..

